# Two‐Year Follow‐Up of a Multidisciplinary Lifestyle Intervention for Rheumatoid Arthritis and Osteoarthritis

**DOI:** 10.1002/acr.25553

**Published:** 2025-06-04

**Authors:** Carlijn A. Wagenaar, Wendy Walrabenstein, Marike van der Leeden, Franktien Turkstra, Martijn Gerritsen, Jos W. R. Twisk, Maarten Boers, Martin van der Esch, Henriët van Middendorp, Peter J. M. Weijs, Dirkjan van Schaardenburg

**Affiliations:** ^1^ Reade Center for Rheumatology and Rehabilitation Amsterdam University Medical Centers, Universiteit van Amsterdam, and Amsterdam Rheumatology and Immunology Center Amsterdam The Netherlands; ^2^ Reade Center for Rheumatology and Rehabilitation Amsterdam University Medical Centers, Vrije Universiteit, and Amsterdam Movement Sciences Research Institute Amsterdam The Netherlands; ^3^ Reade Center for Rheumatology and Rehabilitation Amsterdam The Netherlands; ^4^ Amsterdam University Medical Centers Vrije Universiteit Amsterdam The Netherlands; ^5^ Reade Center for Rheumatology and Rehabilitation and Amsterdam University of Applied Sciences Amsterdam The Netherlands; ^6^ Leiden University Leiden The Netherlands; ^7^ Amsterdam University Medical Centers Vrije Universiteit and Amsterdam University of Applied Sciences Amsterdam The Netherlands

## Abstract

**Objective:**

The Plants for Joints (PFJ) intervention, including a whole‐food plant‐based diet, exercise, and stress reduction, reduced signs and symptoms of rheumatoid arthritis (RA) or metabolic syndrome–associated hip or knee osteoarthritis (MSOA) compared to usual care. This study aimed to examine outcomes two years after the PFJ intervention.

**Methods:**

After two 16‐week randomized controlled trials in people with (1) RA or (2) MSOA, control groups received the active PFJ intervention. All participants were then observed in a two‐year observational extension study. Primary outcomes were Disease Activity Score in 28 joints (DAS28) (RA) and Western Ontario and McMaster Universities Osteoarthritis Index (WOMAC) (MSOA). Secondary outcomes included body composition, metabolic outcomes, medication changes, and adherence to intervention recommendations. Within‐group differences were assessed using linear mixed models, comparing the start and end of the intervention to two years after intervention.

**Results:**

A total of 48 of 77 participants with RA (62%) and 44 of 64 participants with MSOA (69%) completed the extension study. Two years after the intervention, the DAS28 in participants with RA (–0.9 points, 95% confidence interval [CI] –1.2 to –0.6 points) and WOMAC score in participants with MSOA (–8.8 points, 95% CI –12.6 to –5.1 points) were significantly lower than start intervention. In addition, C‐reactive protein in the RA group and weight, body mass index, waist circumference, and diastolic blood pressure in the MSOA group were significantly lower compared to start intervention. Primary end points remained similar from the end of the intervention to the end of the extension study. During the extension study, medication use decreased slightly, and participants continued to follow the intervention recommendations.

**Conclusion:**

Two years after the PFJ intervention, improvements in RA disease activity, MSOA symptoms and functioning, and intervention adherence were sustained.

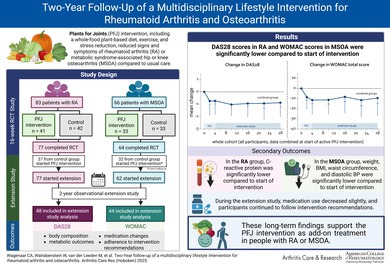

## INTRODUCTION

The Plants for Joints (PFJ) randomized controlled trial investigated the effect of a multidisciplinary lifestyle intervention based on a whole‐food plant‐based diet, physical activity, and stress management in people with low to moderately active rheumatoid arthritis (RA) or metabolic syndrome–associated hip or knee osteoarthritis (MSOA).^1^ After four‐month intervention, participants with RA showed significant disease activity reduction (mean Disease Activity Score in 28 joints [DAS28] –0.9 points),^2^ and participants with MSOA had less pain and stiffness, and improved physical function (mean Western Ontario and McMaster Universities Osteoarthritis Index [WOMAC] score –11 points) compared to a usual care control group.^3^ Both RA and MSOA groups had improved metabolic outcomes, including weight, fat mass, hemoglobin A1c (HbA1c), and low‐density lipoprotein (LDL) cholesterol.^2^, ^3^ After completing the randomized controlled trials, the control groups received the same intervention, and all participants took part in an observational extension study. A year after the PFJ lifestyle intervention, improvements of disease activity and metabolic outcomes within RA and MSOA groups were sustained and related to intervention adherence, with a net decrease of medication.^4^ Because improvements in health behavior and status are not always maintained after a successful lifestyle intervention, all participants were followed up for an additional year. This study aimed to determine disease activity, metabolic health, medication use, and adherence to intervention recommendations two years after intervention in participants with RA and participants with MSOA. Results are presented separately for RA and MSOA but combined in one report, as the same intervention was used.

**SIGNIFICANCE & INNOVATIONS**

- In two randomized controlled trials the 16‐week Plants for Joints (PFJ) multidisciplinary lifestyle intervention significantly improved disease activity or symptoms and metabolic health in people with rheumatoid arthritis (RA) or metabolic syndrome–associated hip or knee osteoarthritis (MSOA).
- After two years, improvements in disease activity (RA), symptoms, and functioning (MSOA) and metabolic outcomes, as well as adherence to intervention recommendations, were largely sustained.
- These long‐term findings support the PFJ intervention as add‐on treatment in people with RA or MSOA.

## MATERIALS AND METHODS

### Design, study sample, and intervention

This study reports the second year of the PFJ extension study; first‐year outcomes were previously published.^4^ The design, study sample, and intervention were previously described.^1^, ^2^, ^3^, ^4^ Briefly, two assessor‐masked open‐label randomized controlled trials compared the effect of a multidisciplinary lifestyle intervention to routine care in people with (1) RA or (2) MSOA between May 2019 and December 2021 at the Reade rehabilitation and rheumatology clinic in Amsterdam, The Netherlands.^1^, ^2^, ^3^ People aged ≥18 years were included if they had (1) RA according to the American College of Rheumatology (ACR)/EULAR 2010 criteria, with 2.6 ≤ DAS28 ≤ 5.1, and stable treatment with or without disease‐modifying antirheumatic drugs for ≥3 months^5^, ^6^ or (2) hip and/or knee osteoarthritis (OA) according to the ACR clinical criteria and metabolic syndrome according to the National Cholesterol Education Program criteria.^7^, ^8^, ^9^ At the start of the intervention, participants received individual intakes with a dietitian and a physical therapist. During the four‐month intervention, mixed groups of participants with RA and participants with MSOA received theoretical and practical education about a calorie‐unrestricted whole‐food plant‐based diet, physical activity, and sleep and stress management during 10 group meetings of 6 to 12 participants.^1^

After completing the randomized controlled trial, control group participants began the lifestyle intervention. Following the active intervention period, all participants were invited to join an extension study with measurements at 6, 12, 18, and 24 months. Participants were encouraged to adhere to the intervention's recommendations and received monthly newsletters and optional bimonthly webinars.^4^ The original trial protocol included a one‐year extension study, but extra resources allowed for a second follow‐up year, requiring additional written informed consent.

The Medical Ethical Committee of the Amsterdam University Medical Centers approved the study protocol (EudraCT number NL66649.048.18), and all participants provided written informed consent. Study protocols were prospectively registered (International Clinical Trial Registry Platform numbers NL7800 and NL7801) and published.^1^ Data will be shared on reasonable request.

### Primary and secondary outcomes

The primary outcome for RA was the mean change in DAS28 from the start and end of the intervention compared to the end of the extension study. DAS28 was assessed by an independent research nurse. The primary outcome for MSOA was the WOMAC total score (range 0–96, best to worst) measured over the same time with digital questionnaires.^10^ Secondary outcomes included components of the primary outcomes, anthropometric, and metabolic outcomes. Adverse events and joint‐replacement surgeries were recorded.

### Medication changes

Medication use was recorded at each measurement, and changes in medication from the start of the intervention to the end of the extension study were classified as “increase,” “stable,” or “decrease.”^4^ Therapeutic injections in MSOA were also recorded. During the extension study, participants with RA and a DAS28 <2.6 received a protocol as a suggested approach to taper antirheumatic medication with their rheumatologist (Supplementary Material S1). Changes in antirheumatic medication intensity were classified by an independent committee according to prespecified criteria.

### Adherence to intervention recommendations

Adherence was assessed at each measurement using an adapted version of the Lifestyle Index Adherence Score, in which a score of 1.0 indicates 100% adherence to program recommendations: attending all 10 meetings during the intervention, doing stress‐reducing activities 6 days/wk for 10 min/day, doing physical activity 5 days/wk for 30 min/day, and having a mean intake of ≥14 g fiber/1,000 kilocalories (kcal) and <10% saturated fatty acids of total kcal/day (energy%).^1^ A score greater than 1.0 reflects higher minutes of stress‐relieving or physical activity, greater fiber intake, and/or lower saturated fat intake. Dietary intake was measured for four days with a validated digital food diary (Mijn Eetmeter).^11^ A two‐day dietary recall was conducted for participants who had difficulty or had not filled in the food diary themselves. Minutes of physical and stress‐reducing activities in the past week were assessed with a digital questionnaire. The intensity and mode of physical activity, as well as webinar attendance during the extension study, were not recorded.

### Statistical analysis

Participants with RA and participants with MSOA were analyzed separately. To estimate the within‐group change over time (start intervention to end extension and end intervention to end extension) in primary and secondary outcomes, linear mixed models were used. In these models, time was treated as a categorical variable using dummy variables, and the intervention and control groups were combined into one cohort, all starting at month 0 (month 0 for the intervention group and month 4 for the control group). To assess the assumptions of the linear mixed models, we examined the normality of residuals using histograms. If assumptions were violated, such as nonnormality, outcomes were log‐transformed before rerunning the models, and within‐group differences were reported as median difference of complete paired values determined with a Wilcoxon test. The linear mixed models, with the ability to handle data missing at random, incorporated all available participant data until the point they were lost to follow‐up, when applicable. Within‐group changes in primary and secondary outcomes for subgroups of extension study completers and dropouts were assessed using linear mixed models. The Wilcoxon test was used to evaluate whether changes in DAS28 or WOMAC differed significantly between completers and dropouts. Medication changes are described with descriptive statistics. Tertiles of the Lifestyle Index Adherence Score were created, and changes in DAS28 or WOMAC per group were summarized descriptively. All analyses were performed with R version 4.3.1 (2023‐06‐16) and *P* values <0.05 were considered statistically significant.

## RESULTS

### RA

A total of 48 of the 77 trial completers (62%) also completed the two‐year follow‐up. A total of 92% of all trial participants were female, with a mean age of 55 (SD 12) years and a mean baseline body mass index (BMI) of 26 (SD 4) (Supplementary Table 1). Twenty‐nine participants withdrew from the extension study (17 participants in year 2), primarily due to busy schedules, the numerous study measurements, or not providing additional permission for the second follow‐up year (Supplementary Figure 1A).

**Figure 1:**
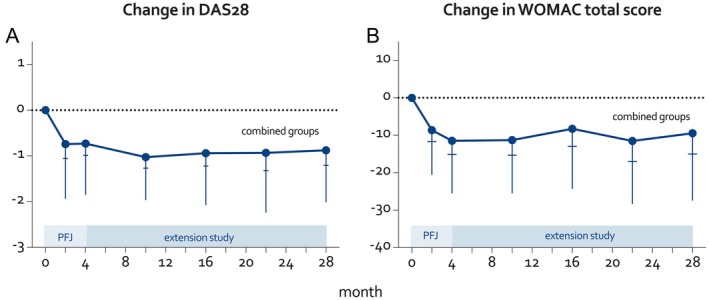
Mean change in DAS28 for (A) participants with rheumatoid arthritis and (B) WOMAC total score for participants with metabolic syndrome–associated hip or knee osteoarthritis for the whole cohort (all participants, data combined at start of active PFJ intervention). Error bars represent 95% confidence intervals (horizontal) and SDs (vertical). *P* values from linear mixed models assessing within‐group differences between the start of the intervention and the end of the extension study are shown. DAS28, Disease Activity Score in 28 joints; PFJ, Plants for Joints; WOMAC, Western Ontario and McMaster Universities Osteoarthritis Index.

Two years after the intervention, DAS28 was significantly lower than at the start: mean –0.9 (95% confidence interval [CI] −1.2 to −0.6, Figure 1A; Supplementary Figure 2A). During the extension study, DAS28 showed a further small, nonsignificant reduction (mean −0.1 [95% CI −0.4 to 0.2]) compared to the end of the intervention (Table 1). Tender joint count and general health components of the DAS28 remained improved two years after the intervention, and there was no longer a significant difference in the erythrocyte sedimentation rate and swollen joint count compared to the start of the intervention (Table 1). Results were similar in participants who completed the two‐year extension study versus those who discontinued prematurely (mean DAS28 change during intervention: completer −0.9, dropout −0.6, *P* = 0.4; mean change up to first‐year extension study: completer −1.0, dropout −0.9, *P* = 0.9; Supplementary Table 2).

**Table 1:** Primary and secondary outcomes for participants with rheumatoid arthritis of the Plants for Joints two‐year extension study*

	Intervention		Extension study		Start intervention to end extension (95% CI)	End intervention to end extension (95% CI)
	Start (n = 77)	End (n = 77)	12 mo (n = 65)	24 mo (n = 48)		
DAS28 and components						
DAS28 ESR, mean (SD)	3.85 (0.86)	3.09 (1.22)	2.84 (1.08)	2.84 (1.14)	−0.9 (−1.2 to −0.6)	−0.1 (−0.4 to 0.2)
DAS28 ESR (seropositive),^a^ mean (SD)	3.88 (0.92)	3.26 (1.29)	2.93 (1.11)	2.90 (1.08)	−0.8 (−1.2 to −0.5)	−0.1 (−0.5 to 0.2)
DAS28 ESR (seronegative),^a^ mean (SD)	3.76 (0.67)	2.62 (0.87)	2.60 (0.98)	2.67 (1.32)	−1.1 (−1.6 to −0.6)	0.0 (−0.5 to 0.6)
Swollen joint count, median (IQR)	1 (0 to 3)	0 (0 to 2)	0 (0 to 1)	1 (0 to 2)	0 (−2 to 1)^b^	1 (0 to 0)^b^
Tender joint count, median (IQR)	3 (1 to 6)	1 (0 to 3)	1 (0 to 3)	0 (0 to 2)	−2 (−2 to −1)	0 (−1 to 0)
General health (VAS), median (IQR)	52 (36 to 64)	26 (10 to 44)	22 (4 to 36)	22 (5 to 46)	−23 (−29 to −16)	−1 (−7 to 6)
ESR, median (IQR), mm/hr	15 (7 to 26)	14 (7 to 27)	12 (5 to 24)	12 (5 to 28)	−2 (−5 to 2)^b^	0 (−4 to 4)^b^
DAS28 ESR <2.6 (%)	–	29 (39)	25 (39)	18 (38)	–	–
DAS28 CRP	2.64 (1.07)	1.84 (1.38)	1.55 (1.25)	1.43 (1.21)	−1.1 (−1.4 to −0.7)	−0.2 (−0.5 to 0.1)
CRP, median (IQR), mg/L	2.4 (1.1 to 5.4)	2.1 (0.7 to 5.2)	1.6 (0.7 to 2.9)	1.3 (0.7 to 3.5)	−1.2 (−2.1 to −0.3)^b^	−0.6 (−1.9 to 0.3)^b^
Serology, median (IQR)						
Rheumatoid factor, kU/L	21.0 (1.2 to 69.0)	14.0 (1.5 to 59.5)	13.5 (1.3 to 39.5)	16.0 (3.1 to 36.0)	−2.0 (−9.6 to −0.9)^b^	−0.3 (−5.3 to 1.6)^b^
ACPA, kU/L	48 (2 to 470)	47 (2 to 605)	73 (2 to 585)	83 (3 to 600)	2 (−18 to 60)^b^	1 (−9 to 23)^b^
Body composition, mean (SD)						
Weight, kg	74.5 (12.9)	71.5 (12.9)	74.6 (13.0)	73.7 (12.6)	0.8 (−0.2 to 1.8)	3.8 (2.9 to 4.8)
BMI, kgm^−2^	26.3 (4.3)	25.2 (4.4)	26.1 (4.3)	25.8 (3.9)	0.3 (−1.0 to 0.6)	1.3 (1.0 to 1.7)
Waist circumference, cm	91.0 (11.2)	87.6 (11.2)	89.8 (11.4)	89.4 (10.7)	−0.4 (−1.8 to 1.0)	3.0 (1.6 to 4.5)
Waist circumference (female participants)^c^	90.2 (11.1)	86.9 (11.1)	89.0 (11.4)	88.3 (10.5)	0.0 (−1.6 to 1.5)	3.3 (1.8 to 4.9)
Waist circumference (male participants)^c^	100.3 (8.4)	96.2 (9.7)	97.3 (8.5)	96.8 (9.4)	−3.5 (−6.0 to −1.0)	0.5 (−1.4 to 2.5)
Metabolic markers						
HbA1c, mean (SD), mmol/mol	36.9 (6.4)	36.0 (6.0)	36.5 (7.0)	37.7 (7.2)	0.6 (−0.1 to 1.2)	1.3 (0.7 to 2.0)
Fasting blood glucose, median (IQR), mmol/L	5.1 (4.8 to 5.4)	4.9 (4.6 to 5.1)	4.9 (4.7 to 5.2)	5.0 (4.7 to 5.3)	−0.1 (−0.3 to 0.1)	0.0 (−0.2 to 0.2)
LDL cholesterol, mean (SD), mmol/L	3.1 (0.9)	2.7 (0.8)	2.9 (0.9)	3.0 (0.9)	0.0 (−0.2 to 0.1)	0.3 (0.2 to 0.5)
HDL cholesterol, mean (SD), mmol/L	1.6 (0.4)	1.6 (0.4)	1.7 (0.4)	1.8 (0.4)	0.1 (0.1 to 0.2)	0.2 (0.1 to 0.3)
Triglycerides, mean (SD), mmol/L	1.1 (0.5)	1.0 (0.4)	1.0 (0.4)	1.0 (0.4)	0.0 (−0.1 to 0.1)^b^	0.0 (−0.1 to 0.1)^b^
Systolic blood pressure, mean (SD), mm Hg	134 (19)	128 (18)	134 (22)	134 (20)	−1 (−5 to 3)	6 (1 to 10)
Diastolic blood pressure, mean (SD), mm Hg	86 (11)	84 (11)	86 (12)	85 (12)	−1 (−4 to 2)	1 (−2 to 5)

* Outcomes from the Plants for Joints cohort at the start and end of the 16‐week intervention period as well as during the two‐year extension study (12 and 24 months after completing the intervention). Within‐group differences are shown between the start and end of the lifestyle intervention and end of the 24‐month follow‐up determined using the linear mixed model when model assumptions were met. ACPA, anti–citrullinated protein antibody; BMI, body mass index; CI, confidence interval; CRP, C‐reactive protein; DAS28, Disease Activity Score in 28 joints; ESR, erythrocyte sedimentation rate; HbA1c, hemoglobin A1c; HDL, high‐density lipoprotein; IQR, interquartile range; LDL, low‐density lipoprotein; VAS, visual analog scale.

^a^ Seropositive, n = 57; seronegative, n = 20.

^b^ Within‐group differences were reported as median difference of complete paired values determined with a Wilcoxon test for outcomes that did not meet model assumptions.

^c^ Female participants, n = 71; male participants, n = 6.

Of the 39 participants who completed the follow‐up and used antirheumatic medication, 17 participants (44%) decreased or stopped medication use (n = 12 decreased and n = 5 stopped, with an average dosage reduction of 58%). Ten participants (26%) maintained stable use, and 12 participants (31%) increased medication (n = 9 added medication, n = 2 switched due to disease activity, and n = 1 had a glucocorticoid injection) (Supplementary Tables 3 and 4). Thirty participants (65%) had improved DAS28 scores (11 with DAS28 <2.6) with stable or less medication compared to baseline. Two years after the intervention, high‐density lipoprotein (HDL) cholesterol was increased, and C‐reactive protein (CRP) levels remained significantly lower compared to the start of the intervention (Table 1). However, weight, BMI, waist circumference, HbA1c, LDL cholesterol, and systolic blood pressure increased during the extension study, although all (except HbA1c) stayed below starting values (Table 1).

### OA

A total of 44 of the 64 trial completers (69%) also completed the two‐year follow‐up. A total of 84% of all trial participants were female, with a mean age of 63 (SD 6) years and a mean baseline BMI of 33 (SD 5) (Supplementary Table 5). Eighteen participants withdrew from the extension study (five in year 2), primarily due to busy schedules, the numerous study measurements, or not providing additional permission for the second follow‐up year (Supplementary Figure 1B).

Two years after the intervention, WOMAC total was significantly lower than at the start: mean −8.8 (95% CI −12.6 to −5.1, Figure 1B; Supplementary Figure 2B). No significant change in WOMAC score was observed between the end of the intervention and the end of the extension study (mean 2.6 [95% CI −0.9 to 6.2]) (Table 2). Furthermore, all components of the WOMAC were significantly improved two years after intervention compared to the start of the intervention (Table 2). Results were similar in participants who completed the two‐year extension study versus those who discontinued prematurely (mean WOMAC total change during intervention: completer −12.0, dropout −10.0, *P* = 0.6; mean change up to first‐year extension study: completer −8.5, dropout −4.3, *P* = 0.7; Supplementary Table 2).

**Table 2:** Primary and secondary outcomes for participants with osteoarthritis of the Plants for Joints two‐year extension study*

	Intervention		Extension study		Start intervention to end extension (95% CI)	End intervention to end extension (95% CI)
	Start (n = 64)	End (n = 62)	12 mo (n = 49)	24 mo (n = 44)		
WOMAC score, mean (SD)						
WOMAC total (0–96)	38.2 (16.2)	26.9 (18.9)	30.4 (18.6)	27.0 (18.8)	–8.8 (–12.6 to –5.1)	2.6 (–0.9 to 6.2)
WOMAC pain (0–20)	7.4 (3.0)	5.1 (3.7)	5.9 (3.7)	4.9 (3.8)	–2.2 (–3.1 to –1.4)	0.1 (–0.7 to 0.9)
WOMAC stiffness (0–8)	4.0 (1.8)	3.0 (2.0)	3.5 (2.2)	3.3 (1.8)	–0.6 (–1.0 to –0.1)	0.5 (0.0 to 1.0)
WOMAC function (0–68)	26.8 (12.8)	18.9 (14.0)	21.1 (13.7)	18.9 (14.0)	–6.3 (–8.9 to –3.3)	1.9 (–0.8 to 4.6)
Inflammation, median (IQR)						
C‐reactive protein, mg/L	1.9 (1.0 to 4.5)	1.3 (0.8 to 3.0)	1.4 (0.9 to 3.3)	1.4 (0.9 to 3.1)	–0.3 (–1.0 to 0.6)^a^	0.3 (0.0 to 0.0)^a^
Body composition, mean (SD)						
Weight, kg	94.9 (15.9)	90.2 (14.9)	90.7 (13.2)	92.1 (12.8)	–3.8 (–5.5 to –2.1)	1.5 (–0.2 to 3.2)
BMI, kgm^−2^	33.3 (5.3)	31.7 (5.0)	31.5 (3.9)	32.3 (4.7)	–1.3 (–1.8 to –0.7)	0.6 (0.0 to 1.1)
Waist circumference, cm	110.0 (12.9)	104.6 (12.3)	105.7 (11.5)	106.7 (9.0)	–3.8 (–5.8 to –1.7)	2.2 (0.4 to 4.1)
Waist circumference (female participants)^b^	108.9 (13.3)	103.3 (12.5)	107.2 (11.8)	105.8 (8.7)	–3.3 (–5.5 to –1.1)	2.7 (0.6 to 4.8)
Waist circumference (male participants)^b^	116.0 (8.9)	112.6 (7.7)	108.8 (10.2)	112.2 (9.4)	–6.3 (–11.8 to –0.8)	–0.5 (–5.0 to 4.1)
Metabolic markers						
HbA1c, mean (SD), mmol/mol	42.6 (8.4)	40.3 (7.2)	40.2 (7.5)	41.0 (6.7)	–0.7 (–1.5 to 0.2)	1.6 (0.6 to 2.5)
Fasting blood glucose, median (IQR), mmol/L	5.8 (5.3 to 6.5)	5.5 (5.1 to 6.2)	5.4 (5.1 to 5.9)	5.7 (5.0 to 6.3)	–0.2 (–0.4 to 0.0)	0.3 (0.0 to 0.5)
LDL cholesterol, mean (SD), mmol/L	3.6 (1.3)	3.3 (1.2)	3.3 (1.4)	3.5 (1.0)	–0.1 (–0.3 to 0.1)	0.2 (–0.1 to 0.4)
HDL cholesterol, mean (SD), mmol/L	1.4 (0.4)	1.4 (0.4)	1.4 (0.4)	1.4 (0.4)	0.0 (0.0 to 0.1)	0.0 (0.0 to 0.1)
Triglycerides, median (IQR), mmol/L	1.6 (1.2 to 2.2)	1.6 (1.0 to 2.1)	1.5 (1.1 to 2.1)	1.5 (1.0 to 1.9)	−0.1 (−0.4 to 0.0)^a^	0.4 (−0.2 to 0.2)^a^
Systolic blood pressure, mean (SD), mm Hg	145 (18)	144 (19)	142 (16)	140 (15)	−5 (−10 to 1)	–4 (−10 to 1)
Diastolic blood pressure, mean (SD), mm Hg	91 (11)	89 (11)	86 (8)	85 (9)	−5 (−8 to −3)	−4 (−6 to −1)

* Outcomes from the Plants for Joints cohort at start and end of the 16‐week intervention period as well as during the two‐year extension study (12 and 24 months after completing the intervention). Within‐group difference shown between the start and end of the lifestyle intervention and end of the 24‐month follow‐up determined using the linear mixed model when model assumptions were met. BMI, body mass index; CI, confidence interval; HbA1c, hemoglobin A1c; HDL, high‐density lipoprotein; IQR, interquartile range; LDL, low‐density lipoprotein; WOMAC, Western Ontario and McMaster Universities Osteoarthritis Index.

^a^ Within‐group differences were reported as median difference of complete paired values determined with a Wilcoxon test for outcomes that did not meet model assumptions.

^b^ Female participants, n = 54; male participants, n = 10.

Two years after the intervention, weight, waist circumference, and diastolic blood pressure remained significantly lower than at the start (Table 2). However, BMI, waist circumference, HbA1c, and fasting blood glucose levels increased during the extension study but stayed below starting values (Table 2). Of the 19 participants who completed the extension study and used pain medication, 10 participants (53%) decreased or stopped, whereas 9 patients (47%) had increased pain medication (Supplementary Table 3). Furthermore, of those who completed the follow‐up and used lipid‐lowering medication, seven participants (44%) decreased, six participants (38%) remained stable, and three participants (19%) increased their medication. During the second year of the extension study, one participant received a hyaluronic acid injection in the knee, and another had knee replacement surgery; both remained in the study. Adverse events for the second year of the extension study for RA and MSOA are described in Supplementary Table 6. Adverse events in the second year were uncommon and mostly mild, with a few moderate events (flu) and two severe events (colon carcinoma and pyelonephritis).

### Adherence to intervention recommendations

Adherence was largely sustained during the extension study: RA Lifestyle Index Adherence Score declined slightly from 1.05 (53% of participants had a score ≥1; end intervention) to 0.99 (45% of participants; end extension study), and MSOA score 1.02 (53%) to 0.99 (45%), respectively (Supplementary Tables 7 and 8). Participants with an adherence score ≥1 at the end of the two‐year extension study showed a trend toward greater changes in DAS28 or WOMAC total scores from the start of intervention to the end of the extension study compared to those with scores <1 (Supplementary Table 9). Two years after the intervention, the median intake of saturated fat (9 energy%, recommendation <10%), fiber (19 g/1,000 kcal, recommendation ≥14 g/1,000 kcal), and time spent on physical activity (193 min/wk, recommendation ≥150 min/wk) were compliant with recommendations in both groups. Time spent per week on stress‐relieving activities remained relatively stable throughout the extension study (36–31 min/wk, recommendation ≥60 min/wk) (Supplementary Tables 7 and 8).

## DISCUSSION

Two years after the intervention, DAS28 in participants with RA and WOMAC in participants with MSOA remained significantly lower than at the start, surpassing the minimal clinically important difference of 0.8 (based on the inclusion criteria) for RA and 20% for pain and physical function for MSOA.^12^, ^13^ The (already low) erythrocyte sedimentation rate and swollen joint count in participants with RA did not remain significantly lower, possibly due to the reduced sample size. Primary outcomes in both groups remained stable during the extension period. These results were achieved despite 44% of participants with RA and 53% of participants with MSOA reducing or stopping antirheumatic or pain medication, respectively.

At the end of the two‐year extension study, participants with RA showed significant improvements in CRP and HDL cholesterol, whereas participants with MSOA had significant reductions in weight, BMI, waist circumference, and diastolic blood pressure compared to the start of intervention. Sustained weight loss and improved waist circumference are notable, as maintaining weight loss over time is typically difficult, and most individuals tend to regain more than half of the lost weight after two years.^14^ During the extension study, weight, BMI, waist circumference, HbA1c, and LDL cholesterol in participants with RA and BMI, waist circumference, HbA1c, and fasting blood glucose levels in participants with MSOA increased, but remained below starting values. This could be due to lower adherence; although our adherence data do not support this, potential underreporting cannot be dismissed.

Lifestyle interventions for RA and OA are clinically relevant as adjunct therapies, helping to reduce disease activity, manage symptoms, and prevent comorbidities. However, their implementation is challenging because of limited access, motivation, and time and cost constraints. The group‐based approach of our intervention, focused on lifestyle education rather than intensive, individualized care, is a key strength and shows strong potential for real‐world clinical implementation. Although few studies report long‐term follow‐up, this study demonstrates sustained benefits, with key factors including social support, an enthusiastic and knowledgeable team, increased health awareness, and motivation from positive effects.^15^ The intervention's emphasis on consistency over perfection enables participants to integrate sustainable habits and recover from setbacks.

Strengths of the study include the long‐term assessment of effectiveness, medication changes, and adherence and the inclusion of only participants with (low to moderately) active RA. Limitations include the lack of a control group, >30% loss to follow‐up, and unmonitored cointerventions such as physical activity or other lifestyle programs. Self‐reported adherence data are a limitation due to potential recall bias or underreporting, although 24‐hour dietary recalls by dietitians helped mitigate underreporting when food diaries were incomplete or unrealistic. The long‐term effect of the intervention on DAS28, WOMAC, and metabolic outcomes may be overestimated due to data lost from participants who dropped out. Although linear mixed models account for missing data assumed to be missing at random, nonrandom missing data cannot be ruled out, particularly as changes in primary and secondary outcomes were slightly larger in participants who completed the extension study compared to those who dropped out. Conversely, reductions in antirheumatic medication may (partially) offset the intervention effect on DAS28. Lastly, because of the multidisciplinary nature, it is impossible to single out the effect of specific components of the lifestyle intervention. Significant improvements in disease activity in RA and pain, stiffness, and physical function in MSOA observed during the PFJ intervention were observed up to two years after program completion, confirming the durability of lifestyle modifications and their positive effects.

## AUTHOR CONTRIBUTIONS

All authors contributed to at least one of the following manuscript preparation roles: conceptualization AND/OR methodology, software, investigation, formal analysis, data curation, visualization, and validation AND drafting or reviewing/editing the final draft. As corresponding author, Dr Wagenaar confirms that all authors have provided the final approval of the version to be published and takes responsibility for the affirmations regarding article submission (eg, not under consideration by another journal), the integrity of the data presented, and the statements regarding compliance with institutional review board/Declaration of Helsinki requirements.

## Supporting information


**Disclosure form**.

**Supplementary Material S1** Protocol to Taper Medication in the “Plants for Joints” Extension study

**Appendix S1:** Supplementary Information
