# Supplementary material for: Two‐Year Follow‐Up of a Multidisciplinary Lifestyle Intervention for Rheumatoid Arthritis and Osteoarthritis
Source: Arthritis Care Res (Hoboken). 2025 Jun 4;77(9):1141–8. doi: 10.1002/acr.25553 (PMC12371306; doi:10.1002/acr.25553)
Supplement: Supplementary file 3 — Appendix S1: Supplementary Information [file ACR-77-1141-s002.pdf]

Supplementary Table 1. Characteristics of rheumatoid arthritis participants at start intervention

**Characteristic (*n* =)**	77
Age, mean (SD), years	54.8 (12.0)
Female sex, number (%)	71 (92)
Body mass index, mean (SD), kg/m2	26.3 (4.3)
Waist circumference, mean (SD), cm	91.0 (11.2)
Waist circumference, female, *n* = 71	90.1 (11.1)
Waist circumference, male, *n* = 6	100.3 (8.4)
**Rheumatoid arthritis**	
Disease duration, mean (SD), years	9.0 (8.4)
RF positive, number (%)	49 (64)
ACPA positive, number (%)	50 (65)
Seropositive, number (%)	57 (74)
DAS28, mean (SD)	3.85 (0.86)
Erosive disease, number (%)	37 (49)
**Antirheumatic medication, number (%)**	
Methotrexate monotherapy	14 (18)
Methotrexate combination therapy	18 (23)
Other csDMARD monotherapy	4 (5)
Other csDMARD combination therapy	7 (9)
bDMARD monotherapy	11 (14)
tsDMARD monotherapy	3 (4)
Glucocorticoid monotherapy	3 (4)
No medication	17 (22)
**Synthetic DMARD**	50 (63)
Methotrexate	32 (41)
Hydroxychloroquine	7 (9)
Sulfasalazine	7 (9)
Leflunomide	4 (5)
**Biological DMARD**	30 (38)
TNF-α inhibitors	17 (21)
Rituximab	5 (6)
Tocilizumab	3 (4)
Abatacept	2 (3)
**Targeted synthetic DMARD**	3 (4)
Baricitinib	3 (4)
**Other**	16 (21)
Prednisone	13 (16)
**Other medication, number (%)**	
Pain	24 (31)
Antihypertensive	16 (21)
Cholesterol-lowering	8 (10)
Glucose-lowering	3 (4)

RF = rheumatoid factor, ACPA = anti–citrullinated protein antibodies, Seropositive = positive for RF or ACPA, DAS28 = 28-joint disease activity score, DMARD = disease-modifying anti-rheumatic drug, csDMARD = conventional synthetic DMARD, bDMARD = biological DMARD, tsDMARD = targeted synthetic DMARD. Methotrexate or cdDMARD combination therapy is the combination of methotrexate or other csDMARDS with any other types of anti-rheumatic drugs. TNF-α inhibitors (Tumor Necrosis Factor alpha) include adalimumab, etanercept, golimumab. Medications used are shown as part of mono- or combination therapy.

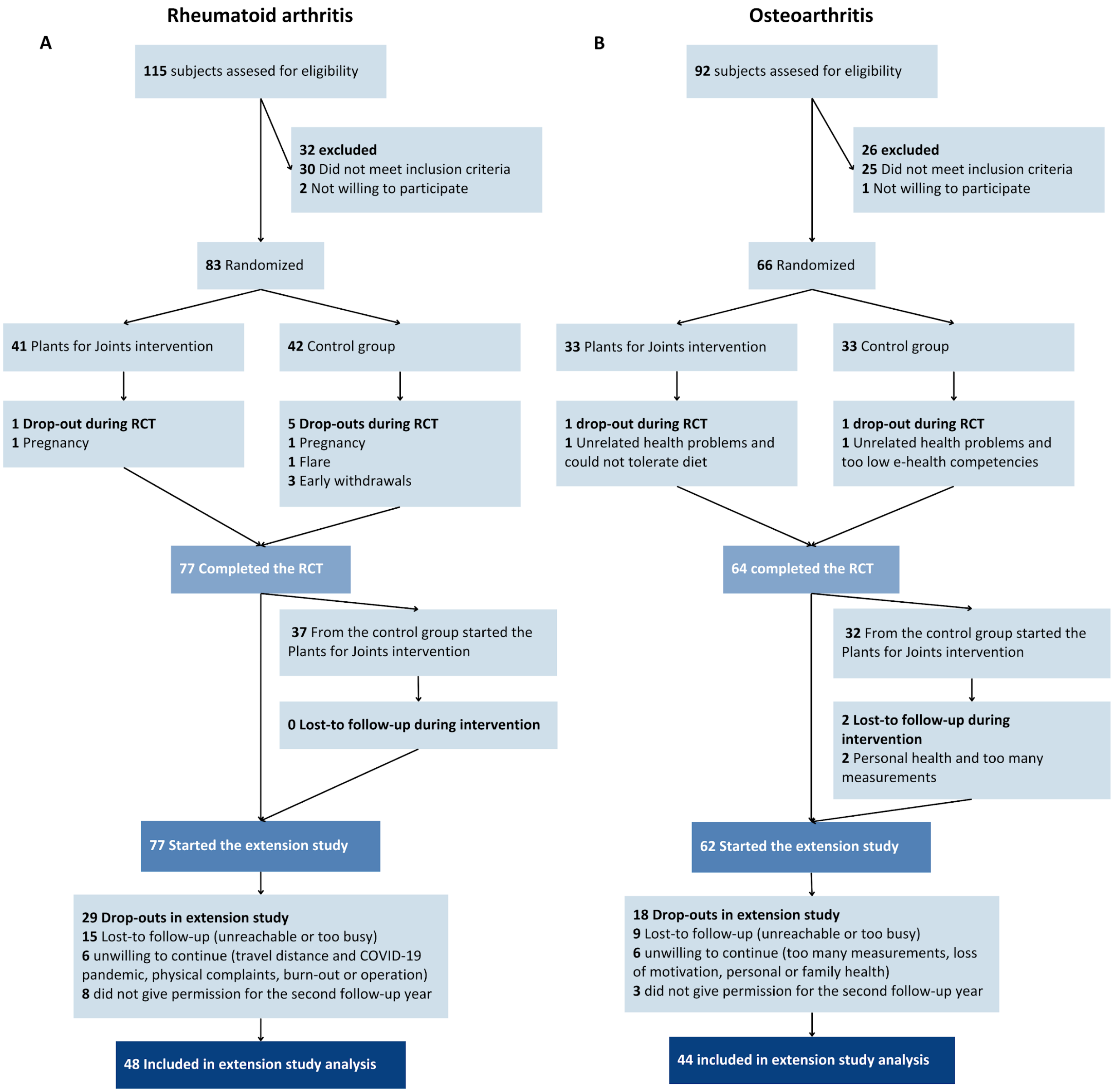

Supplementary Figure 1. Plants for Joints two-year extension study STROBE diagram

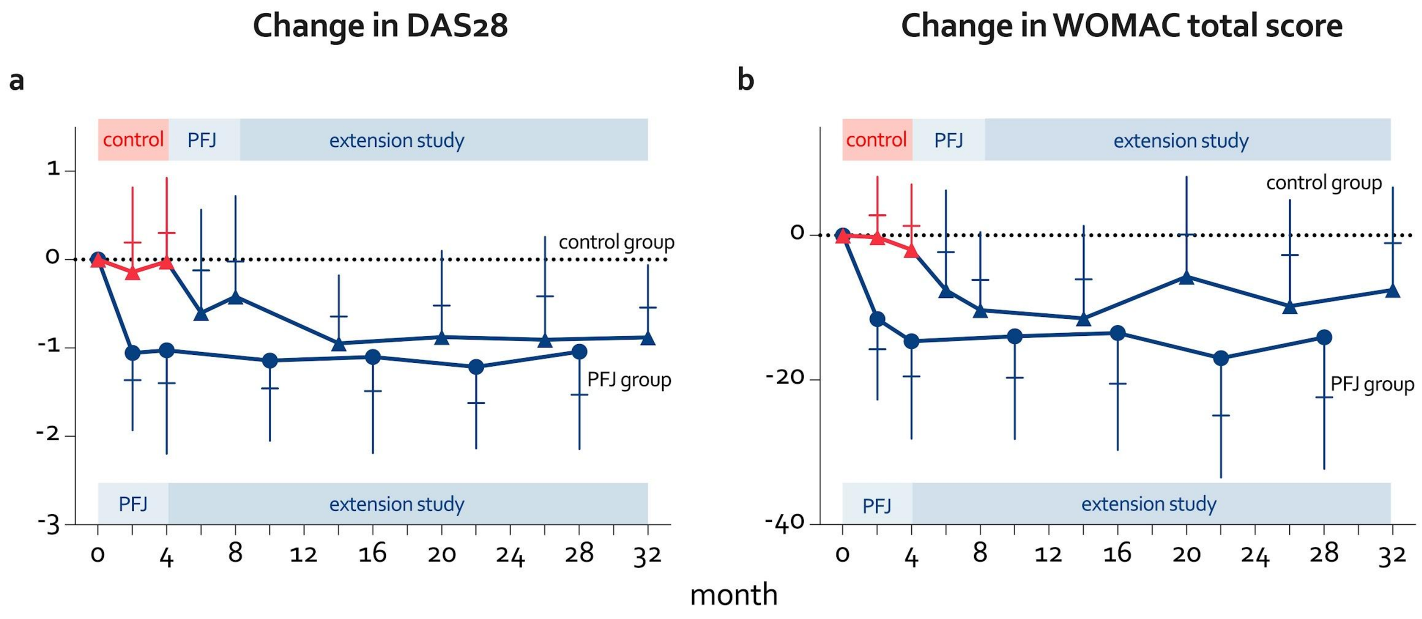

Supplementary Figure 2. Mean change in DAS28 for RA participants (a) and WOMAC total score for MSOA participants (b) presented separately for the original randomized groups (control group who recieved the PFJ intervention after a 4-month waiting period (shown in red), or the PFJ group who recieved the PFJ intervention right away). Of the RA trial completers randomized to the intervention group (n = 40), 22 (55%) completed the two-year extension study, while in the control group (n = 37), 26 (70%) completed it. For the OA trial, 22 (68%) of the 32 participants in both the intervention and control groups completed the two-year extension study. PFJ = Plants for Joints lifestyle intervention. Error bars represent 95% confidence interval (horizontal) and standard deviation (vertical).

Supplementary Table 2. Baseline characteristics and within group difference for extension study completers and drop-outs

	**Extension study completer**		**Drop-out**	
	Start intervention	Within group difference	Start intervention	Within group difference
**Rheumatoid arthritis, *n* =**	48	-	29	
Age, mean (SD), years	54 (12)	-	56 (13)	-
Female sex, number (%)	42 (88)	-	6 (12)	-
DAS28 ESR	3.77 (0.90)	–0.89 (–1.17, –0.61)	3.98 (0.78)	–0.55 (–1.00, –0.11)
Weight (kg)	73.3 (11.9)	–3.3 (–4.0 , –2.5)	76.5 (14.4)	–2.2 (–3.5, –1.0)
Body mass index, mean (SD), kg/m2	25.7 (3.8)	–1.2 (–1.4, –0.9)	27.1 (5.0)	–0.8 (–1.2. –0.3)
Waist circumference, mean (SD), cm	90.1 (11.1)	–4.0 (–5.3, –2.7)	92.5 (11.4)	–2.1 (–3.9, –0.3)
C-reactive protein (mg/l)*	2.3 (1.0 - 5.5)	–0.3 (–1.0, 1.1)	2.4 (1.3 - 4.7)	–0.4 (–1.0, 0.4)
HbA1c (mmol/mol)	37.1 (7.3)	–0.7 (–1.3, –0.2)	36.5 (4.7)	–0.8 (–1.4, –0.2)
LDL-cholesterol (mmol/l)	3.1 (1.0)	–0.4 (–0.6, –0.3)	3.1 (0.9)	–0.3 (–0.4, –0.1)
**Osteoarthritis, *n* =**	44		20	
Age, mean (SD), years	64 (7)		63 (6)	
Female sex, number (%)	38 (86)		16 (80)	
WOMAC total (0-96)	37.1 (15.5)	–12.0 (–15.6, –8.3)	40.7 (17.8)	–10.0 (–16.1, –3.9)
Weight (kg)	95.6 (14.7)	–5.4 (–6.5, –4.4)	93.3 (18.6)	–4.3 (–6.0, –2.7)
Body mass index, mean (SD), kg/m2	33.4 (4.8)	–1.9 (–2.2, –1.5)	32.9 (6.4)	–1.5 (–2.1, –0.9)
Waist circumference, mean (SD), cm	110.4 (11.6)	–6.7 (–8.6, –4.9)	109.2 (15.7)	–3.4 (–6.2, –0.7)
C-reactive protein (mg/l)*	1.9 (1.0 - 4.1)	–0.9 (–1.5, –0.4)	1.9 (1.2 - 4.6)	–0.9 (–2.1, 0.5)
HbA1c (mmol/mol)	41.1 (6.4)	–2.1 (–2.8, –1.5)	45.8 (11.2)	–2.6 (–4.5, –0.7)
LDL-cholesterol (mmol/l)	3.6 (1.1)	–0.3 (–0.5, –0.1)	3.5 (1.7)	–0.2 (–0.5, 0.1)

Baseline characteristics and within group change for subgroups of participants who completed the two-year extension study and participants who dropped-out during the two-year extension study. Continuous variables are reported as mean (SD) when normally distributed or as median (IQR) when skewed. Within-group difference shown between start of the lifestyle intervention and end of the 16-week intervention determined using linear-mixed models. *a linear-mixed model was performed after log transformation and within group differences were reported as median difference of complete paired values determined with a Wilcoxon test. DAS28, 28-joint Disease Activity Score; ESR, erythrocyte sedimentation rate; HbA1c, haemoglobin A1c; LDL, low-density lipoprotein.

Supplementary Table 3. Medication changes for rheumatoid arthritis and osteoarthritis participants after the two-year Plants for Joints extension study

Change within medication groups	Rheumatoid Arthritis, *n* (%)	Osteoarthritis, *n* (%)
**Antirheumatic**	**39 (81)**	**N/A**
Medication increase	12 (31)	
Increase dosage	0 (0)	
Added medication	9 (23)	
Switch (disease activity)	2 (5)	
≥1 glucocorticoid injection	1 (3)	
Stable medication	10 (26)	
No change	9 (23)	
Switch (side-effects)	1 (3)	
Medication decrease	17 (44)	
Decrease dosage	12 (31)	
Stop medication	5 (13)	
**Pain**	**19 (40)**	**19 (43)**
Medication increase	8 (42)	9 (47)
Increase dosage	0 (0)	0 (0)
Added medication	7 (37)	5 (26)
Switch (disease activity)	1 (5)	4 (21)
Stable medication	1 (5)	0 (0)
No change	1 (5)	0 (0)
Switch (side-effects)	0 (0)	0 (0)
Medication decrease	10 (53)	10 (53)
Decrease dosage	3 (16)	1 (5)
Stop medication	7 (37)	9 (47)
**Cholesterol-lowering**	**5 (10)**	**16 (36)**
Medication increase	1 (20)	3 (19)
Increase dosage	0 (0)	0 (0)
Added medication	1 (20)	2 (13)
Switch (disease activity)	0 (0)	1 (6)
Stable medication	2 (40)	6 (38)
No change	2 (40)	5 (31)
Switch (side-effects)	0 (0)	1 (6)
Medication decrease	2 (40)	7 (44)
Decrease dosage	2 (40)	2 (13)
Stop medication	0 (0)	5 (31)
**Antihypertensive**	**11 (23)**	**27 (61)**
Medication increase	5 (45)	7 (26)
Increase dosage	0 (0)	3 (11)
Added medication	5 (45)	2 (7)
Switch (disease activity)	0 (0)	2 (7)
Stable medication	3 (27)	14 (52)
No change	3 (27)	13 (48)
Switch (side-effects)	0 (0)	1 (4)
Medication decrease	3 (27)	6 (22)
Decrease dosage	0 (0)	4 (15)
Stop medication	3 (27)	2 (7)
**Glucose-lowering**	**2 (4)**	**8 (18)**
Medication increase	0 (0)	3 (38)
Increase dosage	0 (0)	2 (25)
Added medication	0 (0)	1 (13)
Switch (disease activity)	0 (0)	0 (0)
Stable medication	1 (50)	2 (25)
No change	1 (50)	2 (25)
Switch (side-effects)	0 (0)	0 (0)
Medication decrease	1 (50)	3 (38)
Decrease dosage	0 (0)	3 (38)
Stop medication	1 (50)	0 (0)

In the RA group, those with stable or reduced medication had a mean change in DAS28 from the start of the intervention to the end of the extension study of –1.02 (1.0), which was slightly smaller than the change in those with increased medication (–1.13 (1.16)), though the difference was not significant ($p = 0.8$). In the MSOA group, those with stable or reduced pain medication showed a trend toward greater improvement in WOMAC total score (–19.7 (19.7)) compared to those with increased pain medication (–2.1 (23.8)), though this difference was also not significant ($p = 0.12$). These results suggest that changes in medication had minimal impact on the observed outcomes.

Supplementary Table 4. Increased and decreased antirheumatic medication use for rheumatoid arthritis participants at the end of the two-year Plants for Joints extension study

- **Increase**
  - **Switch medication (disease activity)**
    - Etanercept 50 mg/week switch to filgotinib 100 mg/day, prednisone 7.5 mg/day added
    - Etanercept 50 mg/week switch to tocilizumab 162 mg/week, prednisone 15 mg/day stopped, methotrexate 7.5 mg/week added, glucocorticoid injection (i.m)
  - **Added medication**
    - Prednisone 7.5 mg/day started, rituximab 1000 mg/6 months to 1000 mg/18 months
    - Adalumimab 40 mg/2 weeks added, methotrexate 10 mg/week started, prednisone 5.0 mg/day stopped, Glucocorticoid injection (i.m.)
    - Methotrexate 25 mg/week to 12.5mg/week, adalumimab 40 mg/2 weeks started
    - Methotrexate 7.5 mg/week started
    - Methotrexate 15 mg/week started, leflunomide 10 mg/day stopped, prednisone 2.5 mg/day to 5 mg/day
    - Adalumimab 40 mg/2 weeks started, methorexate 25 mg/week switch to leflunomide 20 mg/day, prednisone 10 mg/day to 5 mg/day, glucocorticoid injection (i.m.)
    - Leflunomide 20 mg/day started
    - Methotrexate 12.5 mg/week started
    - Sulfasalazine 2000 mg/day started
  - **≥ 1 Glucocorticoid injection**
    - Glucorticoid injection (i.m.), adalumimab 20 mg/week stopped
- **Decrease**
  - **Decrease dosage / increase interval**
    - Methotrexate 20 mg/week to 15 mg/week
    - Etanercept 50 mg/week to 50 mg/10 days
    - Sulfasalazine 1500 mg/day to 1000 mg/day
    - Rituximab 500 mg/6 months to 200 mg/6 months
    - Methotrexate 25 mg/week to 12.5 mg/week
    - Leflunomide 20 mg/day to 10 mg/day
    - Methotrexate 15 mg/day to 5 mg/day, tocilizumab (s.c.) 162 mg/week to 162 mg/2 weeks
    - Leflunomide 20 mg/day to 10 mg/day
    - Methotrexate 20 mg/week to 15 mg/week
    - Etanercept 50 mg/week to 50 mg/2 weeks
    - Etanercept 50 mg/week to 50 mg/2 weeks
    - Sulfasalazine 2000 mg/day to 1500 mg/day
  - **Stopped one medication**
    - Hydroxychloroquine 200 mg/day stopped, Methotrexate 10 mg/week to 5 mg/week
  - **Stopped all anti-rheumatic medication**
    - Leflunomide 20 mg/day stopped
    - Prednisone 7.5 mg/day stopped
    - Abatacept (i.v.) 750 mg/4 weeks stopped, methotrexate 10 mg/week stopped
    - Methotrexate 7.5 mg/week stopped

Changes shown between start of the intervention and at the end of the two-year extenstion study. i.m. = intra-muscular injection, i.a. = intra-articular injection, i.v = intra-venous, s.c. = subcutaneous injection.

Supplementary Table 5. Characteristics of osteoarthritis participants at start intervention

**Characteristic (*n* =)**	64
Age, mean (SD), years	63.5 (6.4)
Female sex, number (%)	54 (84)
Body mass index, mean (SD), kg/m2	33.3 (5.3)
Weight, mean (SD), kg	94.9 (15.9)
Waist circumference, mean (SD), cm	110.0 (12.9)
Waist circumference, female, *n* = 54	108.9 (13.3)
Waist circumference, male, *n* = 10	116.0 (8.9)
**Location OA, number (%)**	
Knee OA	25 (39)
Hip OA	12 (19)
Knee and hip OA	27 (42)
**Kellgren Lawrence grade, number (%)**	
OA knee grade 2-4	47 (73)
OA hip grade 2-4	50 (78)
**WOMAC score, mean (SD)**	
WOMAC total (range 0 - 96)	38.2 (16.2)
WOMAC pain (range 0 - 20)	7.4 (3.0)
WOMAC stiffness (range 0 - 8)	4.0 (1.8)
WOMAC physical function (0 - 68)	26.8 (12.8)
**Metabolic syndrome-associated co-morbidities, number (%)**	
Hypertension	54 (82)
(Pre)diabetes type 2	12 (19)
Hyperlipidemia	45 (70)
**Medication use, number (%)**	
Pain	24 (38)
Antihypertensive	41 (64)
Cholesterol-lowering	24 (38)
Glucose-lowering	13 (20)

OA grade classified by Kellgren and Lawrence system for classification of osteoarthritis. WOMAC = The Western Ontario and McMaster Universities Osteoarthritis Index.

Supplementary Table 6. Adverse events for participants with rheumatoid arthritis or osteoarthritis during the second year of the Plants for Joints extension study

Type	Time point	Description	Serious AE	Severity	Related to the intervention
**Rheumatoid arthritis**					
Other	Extension study 18 m	Coughing with sputum production	No	Mild	No
Other	Extension study 18 m	Hairloss	No	Mild	Unlikely
Other	Extension study 24 m	Rosacea	No	Mild	Unlikely
Other	Extension study 24 m	Diagnosis polymyalgia rheumatica	No	Moderate	Unlikely
Other	Extension study 24 m	Back hernia	No	Moderate	Unlikely
Infection	Extension study 24 m	Pyelonefritis with kidney stones	Yes	Severe	Unlikely
Other	Extension study 24 m	Ganglion left wrist	No	Moderate	Unlikely
Abdominal complaints	Extension study 24 m	Gastric reflux	No	Mild	Unlikely
**Osteoarthritis**					
Abdominal complaints	Extension study 18 m	Constipation	No	Mild	Unlikely
Abdominal complaints	Extension study 18 m	Gall bladder colic	No	Mild	Unlikely
Other	Extension study 18 m	Sprained ankle	No	Mild	Unlikely
Other	Extension study 18 m	Fell	No	Mild	Unlikely
Other	Extension study 18 m	Depressive thoughts	No	Mild	Unlikely
Abdominal complaints	Extension study 24 m	Gastric reflux	No	Mild	Unlikely
Other	Extension study 24 m	Hairloss	No	Mild	Unlikely
Other	Extension study 24 m	Dizziness and heart palpitations	No	Mild	Unlikely
Other	Extension study 24 m	Hairloss	No	Mild	Unlikely
Other	Extension study 24 m	Fell on stairs	No	Mild	Unlikely
Infection	Extension study 24 m	Flu	No	Moderate	Unlikely
Other	Extension study 24 m	Colon cancer diagnosis	Yes	Severe	Unlikely
Infection	Extension study 24 m	Flu	No	Moderate	Unlikely

Adverse events during the intervention and first year of the extension study were previously pusblished (Walrabenstein et al. Rheumatology (2023), Walrabenstein et al. Osteoarthrtis and Cartilage (2023), Wagenaar et al. RMD Open (2024))

Supplementary Table 7. Lifestyle descriptives for rheumatoid arthritis participants in the Plants for Joints two-year extension study

	**Intervention**			**Extension study**	
	**Start**	**Halfway**	**End**	**12 months**	**24 months**
**Diet characteristics, *n***	65	56	49	49	43
Energy, kcal	1737 (324)	1686 (342)	1706 (332)	1833 (282)	1747 (343)
Fat, g	71.7 (62.3 - 81.0)	73.1 (56.9 - 80.4)	71.0 (59.6 - 81.4)	81.5 (64.8 - 91.4)	73.9 (59.8 - 82.8)
Fat, energy%	37.2 (5.9)	38.0 (7.0)	36.6 (5.5)	38.5 (5.6)	36.8 (6.2)
Saturated fat, g	23.0 (16.4 - 28.3)	13.9 (11.3 - 17.1)	14.0 (10.5 - 18.6)	18.5 (14.9 - 24.2)	16.2 (13.0 - 19.7)
Saturated fat, energy%	11.8 (9.7 - 14.1)	7.3 (6.2 - 8.9)	7.7 (6.3 - 9.4)	9.1 (7.4 - 11.3)	8.7 (7.4 - 9.7)
Carbohydrate, g	186.3 (42.4)	186.8 (40.3)	186.4 (45.3)	193.9 (38.1)	186.3 (44.7)
Carbohydrate, energy%	43.0 (39.0 - 47.0)	44.0 (41.0 - 48.0)	45.0 (41.0 - 47.0)	44.0 (39.0 - 46.0)	43.6 (39.0 - 45.8)
Protein, g	66.8 (55.3 - 76.3)	55.4 (46.0 - 67.5)	58.1 (49.1 - 64.4)	62.1 (52.8 - 67.7)	56.8 (50.7 - 68.2)
Protein, g/kg body weight*	0.88 (0.74 - 1.11)	0.81 (0.69 - 0.92)	0.85 (0.71 - 1.01)	0.87 (0.79 - 1.02)	0.84 (0.74 - 1.00)
Fiber, g	24.5 (7.8)	36.8 (9.9)	36.1 (9.9)	34.1 (10.9)	33.1 (10.3)
Fiber, g/1000 kcal	14.2 (4.0)	21.9 (4.6)	21.3 (4.7)	18.7 (5.2)	18.8 (4.2)
**Physical activity, *n***	76	72	72	57	38
min/wk	158 (94 - 255)	193 (123 - 298)	193 (120 - 269)	188 (105 - 305)	193 (111 - 289)
**Stress reducing activities, *n***	75	74	73	58	38
min/wk	22 (10 - 55)	33 (20 - 59)	36 (10 - 55)	35 (20 - 63)	31 (13 - 45)
**Lifestyle Index adherence score, *n***			72	58	38
Total score**			1.05 (0.86 - 1.21)	0.99 (0.78 - 1.22)	0.99 (0.85 - 1.18)
score ≥ 1, *n* (%)			38 (53)	29 (47)	17 (45)
Adherence meetings			0.90 (0.90 - 1.00)	-	-
score = 1, *n* (%)			37 (48)	-	-
Adherence exercise			1.34 (1.03 - 1.58)	1.21 (0.63 - 1.69)	1.24 (0.70 - 1.66)
score ≥ 1, *n* (%)			54 (78)	39 (66)	23 (61)
Adherence stress			0.67 (0.17 - 0.93)	0.70 (0.33 - 1.00)	0.53 (0.29 - 0.77)
score ≥ 1, *n* (%)			17 (29)	16 (30)	5 (15)
Adherence diet			1.43 (0.32)	1.25 (0.33)	1.29 (0.27)
score ≥ 1, *n* (%)			44 (94)	38 (78)	36 (84)

Lifestyle descriptives at start and end of the 4-month intervention period as well as during the 2-year extension study (12 and 24 months after completing the intervention). Continuous variables reported as mean (SD) when normally distributed or as median (IQR) when skewed. **A Lifestyle Index adherence score of 1.0 indicates 100% adherence, defined as attendance of all ten meetings during the intervention, stress-reducing activities six days per week for ten minutes per day, physical activity five days per week for 30 minutes per day, and mean intake of ≥14 g fibre per 1000 kilocalories (kcal) and <10% saturated fatty acids of total kcal per day (energy%). kcal = kilocalories, energy% = percentage of total energy in kilocalories. *Adjustment of body weight for participants with a BMI ≥ 30 to match a BMI of 27.5.

Supplementary Table 8. Lifestyle descriptives for osteoarthritis participants in the Plants for Joints two-year extension study

	Intervention			Extension study	
	**Start**	**Halfway**	**End**	**12 months**	**24 months**
**Diet characteristics, *n***	56	41	47	41	39
Energy, kcal	1803 (447)	1699 (270)	1691 (301)	1682 (273)	1729 (368)
Fat, g	74.3 (57.7 - 94.2)	66.5 (60.5 - 76.3)	69.8 (58.7 - 81.7)	68.2 (61.7 - 82.7)	70.7 (60.0 - 87.0)
Fat, energy%	38.0 (33.8 - 41.3)	37.6 (32.5 - 40.6)	37.8 (34.2 - 40.6)	37.3 (35.3 - 41.2)	38.1 (33.0 - 42.4)
Saturated fat, g	24.5 (16.5 - 31.2)	12.7 (10.2 - 16.9)	13.0 (11.1 - 16.1)	14.3 (11.4 - 22.5)	15.2 (11.7 - 43.8)
Saturated fat, energy%	12.6 (9.3 - 14.5)	6.9 (6.2 - 8.6)	7.5 (6.2 - 8.8)	7.6 (6.7 - 10.5)	8.5 (7.2 - 10.9)
Carbohydrate, g	178.0 (41.4)	184.9 (33.8)	174.4 (46.0)	170.0 (44.9)	175.6 (44.0)
Carbohydrate, energy%	39.8 (35.6 - 44.6)	42.7 (39.0 - 47.0)	41.9 (38.9 - 46.1)	42.9 (38.7 - 45.9)	40.9 (37.6 - 45.8)
Protein, g	69.6 (59.5 - 83.7)	56.5 (53.0 - 65.0)	61.3 (52.3 - 69.2)	58.6 (52.8 - 69.5)	64.8 (52.7 - 77.7)
Protein, g/kg body weight*	0.90 (0.76 - 1.07)	0.74 (0.65 - 0.85)	0.79 (0.67 - 0.86)	0.78 (0.67 - 0.93)	0.85 (0.69 - 0.94)
Fiber, g	22.9 (19.2 - 32.2)	36.1 (31.8 - 42.2)	33.1 (29.5 - 42.3)	33.2 (25.3 - 40.0)	30.8 (25.4 - 37.1)
Fiber, g/1000 kcal	12.9 (11.3 - 17.2)	21.4 (19.0 - 24.8)	20.5 (17.3 - 23.9)	21.1 (15.6 - 23.5)	18.7 (14.1 - 22.0)
**Physical activity, *n***	62	58	58	47	42
min/wk	190 (101 - 279)	181 (122 - 287)	189 (111 - 303)	195 (144 - 333)	193 (124 - 289)
**Stress reducing activities, *n***	61	57	56	47	42
min/wk	33 (0 - 55)	44 (15 - 60)	33 (9 - 62)	35 (8 - 66)	33 (0 - 55)
**Lifestyle Index adherence score, *n***			58	47	42
Total score**			1.02 (0.82 - 1.23)	1.08 (0.88 - 1.27)	0.99 (0.81 - 1.19)
score ≥ 1, *n* (%)			31 (53)	29 (58)	19 (45)
Adherence meetings			0.90 (0.90 - 1.00)	-	-
score = 1, *n* (%)			30 (48)	-	-
Adherence exercise			1.19 (0.88 - 1.69)	1.34 (1.01 - 1.81)	1.22 (0.88 - 1.52)
score ≥ 1, *n* (%)			37 (64)	37 (74)	28 (66)
Adherence stress			0.59 (0.16 - 1.01)	0.67 (0.15 - 1.05)	0.53 (0.00 - 0.88)
score ≥ 1, *n* (%)			15 (34)	15 (37)	10 (33)
Adherence diet			1.47 (1.22 - 1.62)	1.50 (1.06 - 1.62)	1.35 (0.99 - 1.48)
score ≥ 1, *n* (%)			43 (91)	32 (78)	29 (74)

Lifestyle descriptives at start and end of the 4-month intervention period as well as during the 2-year extension study (12 and 24 months after completing the intervention). Continuous variables reported as mean (SD) when normally distributed or as median (IQR) when skewed. **A Lifestyle Index adherence score of 1.0 indicates 100% adherence, defined as attendance of all ten meetings during the intervention, stress-reducing activities six days per week for ten minutes per day, physical activity five days per week for 30 minutes per day, and mean intake of ≥14 g fibre per 1000 kilocalories (kcal) and <10% saturated fatty acids of total kcal per day (energy%). kcal = kilocalories, energy% = percentage of total energy in kilocalories. *Adjustment of body weight for participants with a BMI ≥ 30 to match a BMI of 27.5.

Supplementary Table 9. Subgroups of lifestyle adherence score

	**Lifestyle adherence score tertiles**			**Above or under 1**	
	low	moderate	high	< 1	≥ 1
**Rheumatoid arthritis**					
Total score	–1.0 (–1.8 - –0.4)	–0.5 (–0.9 - –0.2)	–1.0 (–1.6 - –0.4)	–0.6 (–1.3 - –0.1)	–0.9 (–1.4 - –0.5)
Adherence exercise	–0.5(–1.0 - –0.0)	–1.0 (–2.5 - –0.6)	–0.7 (–1.3 - –0.3)	–0.9 (–1.3 - –0.1)	–0.7 (–1.4 - –0.4)
Adherence stress	–0.5 (–1.1 - –0.3)	–1.3 (–1.7 - –0.7)	–0.8 (–1.2 - –0.2)	–0.7 (–1.4 - –0.4)	0.1 (–0.8 - 0.5)
Adherence diet	–0.7 (–1.3 - –0.4)	–0.5 (–1.4 - –0.0)	–1.0 (–1.3 - –0.5)	–0.7 (–0.9 - –0.3)	–0.8 (–1.4 - –0.2)
**Osteoarthritis**					
Total score	–8.0 (–27.8 - –3.3)	–5.0 (–11.5 - –5.4)	–11.0 (–18.3 - –8.4)	–4.0 (–18.5 - 1.5)	–10.0 (–17.5 - –2.0)
Adherence exercise	–6.0 (–25.8 - –2.3)	–10.0 (–18.0 - 0.8)	–7.5 (–13.8 - 2.8)	–6.0 (–25.8 - –2.3)	–8.0 (–16.8 - 2.8)
Adherence stress	–10.0 (–26.3 - –4.3)	–4.0 (–12.0 - 5.0)	–7.0 (–14.0 - 13.5)	–6.5 (–15.0 - 2.8)	–11.0 (–18.25 - –5.5)
Adherence diet	–3.0 (–5.0 - 8.0)	–10.0 (–13.0 - –8.0)	–21.0 (–30.5 - –6.0)	–1.0 (–4.8 - 6.5)	–12.0 (–25.5 - –4.5)

Results show median (IQR) change in DAS28 for rheumatoid arthritis participants or WOMAC score for osteoarthritis participants from the start of the intervention to the end of the two year extension study stratified based on tertile of Lifestyle Index Adherence Score or a Lifestyle Index Adherence Score < or to ≥1. A Lifestyle Index adherence score of 1.0 indicates 100% adherence, defined as attendance of all ten meetings during the intervention, stress-reducing activities six days per week for ten minutes per day, physical activity five days per week for 30 minutes per day, and mean intake of ≥14 g fibre per 1000 kilocalories (kcal) and <10% saturated fatty acids of total kcal per day (energy%). The Lifestyle Index Adherence Score was calculated using adherence data of the lifestyle components from the end of the two-year extension study.
